# Draft genome sequence of multidrug-resistant *Staphylococcus aureus* strain MB02 isolated from the cesarean wound of a 32-year-old pregnant woman in Ogbomoso, Nigeria

**DOI:** 10.1128/mra.01357-25

**Published:** 2026-06-11

**Authors:** Muritala Issa Bale, Wakeel Okanlawon Muritala, Micheal Olubusayo Binuyo, Rasheed Omotayo Adeyemo, Taofeek Tope Adegboyega, Ismail Abiola Adebayo, Adam Moyosore Afodun, Ahmed Adedeji, Saheed Akinola Adekunle

**Affiliations:** 1 Department of Microbiology and Parasitology, School of Medicine and Pharmacy, College of Medicine and Health Sciences, University of Rwanda, Huye, Rwanda; 2 Department of Microbiology, Federal University of Health Sciences, Ila Orangun, Nigeria; 3 Department of Obstetrics and Gynaecology, Ladoke Akintola University of Technology 119059, Ogbomoso, Nigeria; 4 Department of Biochemistry, Molecular Biology and Genetics, College of Medicine and Health Sciences, University of Rwanda, Huye, Rwanda; 5 Department of Medical Imaging Sciences, College of Medicine and Health Sciences, University of Rwanda, Huye, Rwanda; 6 Department of Pharmacology, School of Medicine and Pharmacy, College of Medicine and Health Sciences, University of Rwanda, Huye, Rwanda; University of Pittsburgh School of Medicine, Pittsburgh, Pennsylvania, USA

**Keywords:** multidrug, *Staphylococcus aureus*, cesarean wound

## Abstract

This study reports the draft genome sequence of *Staphylococcus aureus* strain MB02, a multidrug-resistant isolate recovered from a cesarean wound of a 32-year-old pregnant woman in Ogbomoso, Nigeria.

## ANNOUNCEMENT

*Staphylococcus aureus* is an opportunistic pathogen frequently present on the skin and in the nasal passages and a major cause of surgical site infections following cesarean sections (C-sections) (1). C-section wounds provide an ideal entry site due to skin barrier breach and the presence of nutrient-rich tissue fluid. Methicillin-resistant *S. aureus* strains pose particular risks due to resistance to first-line antibiotics, and infections can lead to serious complications, including cellulitis, abscess formation, wound dehiscence, and systemic infection (2, 3).

This genome announcement presents the draft genome sequence of *S. aureus* strain MB02, isolated from a cesarean section of a 32-year-old pregnant woman in Ogbomoso, Nigeria. It provides genomic insights into resistance and virulence genes to support genomic surveillance, antimicrobial stewardship, and pathogen evolution research.

Strain MB02 was isolated in July 2024 from a wound swab collected from a 32-year-old pregnant woman attending Ladoke Akintola University Teaching Hospital, Ogbomoso, Nigeria. The isolate was cultured on mannitol salt agar (Oxoid, England) and incubated aerobically at 37°C for 24 hours. Preliminary identification was based on Gram staining (gram-positive cocci in clusters) and a positive catalase and coagulase test. Antimicrobial susceptibility testing was performed using the Kirby-Bauer disk diffusion method following CLSI guidelines (4), and it revealed resistance to multiple antibiotics, including cefoxitin, indicating methicillin resistance.

The cells of a single colony of verified *Staphylococcus aureus* were extracted by centrifugation at 12,000 × *g* for 10 minutes after it had been cultured overnight in Luria-Bertani broth (Himedia, India) in a shaking incubator at 37°C and 200 rpm. Using the QIAamp DNA Mini Kit (QIAGEN, Germany), genomic DNA was extracted in accordance with the manufacturer’s instructions. DNA concentration was determined using the Qubit dsDNA High Sensitivity Assay Kit (Thermo Fisher Scientific), and DNA integrity was evaluated by 1% (wt/vol) agarose gel electrophoresis. DNA purity was assessed using a NanoDrop ND-1000 spectrophotometer (Thermo Fisher Scientific).

The whole genome was sequenced using Illumina MiSeq technology with 2 × 150 bp paired-end reads. Library preparation was performed using the MiSeq DNA High Throughput Library Prep Kit (Illumina, USA) following the manufacturer’s protocol. Sequencing yielded 3,633,644 paired-end reads (1,816,822 read pairs; 372.6 Mb total bases) deposited in the Sequence Read Archive under accession number SRR32454207. Quality assessment of raw reads was performed using FastQC (version 0.12.1) (5). Adapter sequences and low-quality bases were trimmed using Trimmomatic (version 0.39) (6) with a minimum read length cutoff of 36 bp. *De novo* assembly was performed using SPAdes (version 3.15.3) (7), producing 45 contigs locally. The genome assembly deposited at NCBI (GenBank accession GCA_049154425.1) was processed by NCBI’s pathogen detection pipeline using SKESA (version 2.2), resulting in 76 contigs. Genome annotation was performed using the NCBI Prokaryotic Genome Annotation Pipeline (PGAP) (8).

Genome completeness was assessed using CheckM, which estimated 100% completeness based on lineage-specific single-copy marker genes for the Staphylococcaceae family. In addition to phenotypic identification, genome-based species confirmation was performed by average nucleotide identity (ANI) analysis using NCBI’s taxonomy check pipeline, yielding 99.11% ANI against the *S. aureus*-type strain (GCA_001544175.1), confirming the isolate as *S. aureus*.

Antimicrobial resistance gene analysis was performed using NCBI AMRFinderPlus, which identified resistance determinants: *blaZ*, *blaI*, and *blaR1* (beta-lactam resistance); *erm(C)* (macrolide-lincosamide-streptogramin B resistance); *aadD1* (aminoglycoside resistance); *tet(38*) and *tet(L)* (tetracycline resistance); *mupA* (mupirocin resistance); *fusB* (fusidic acid resistance); *fosB* (fosfomycin resistance); *mepA* (multidrug efflux pump); *bleO* (bleomycin resistance); and the *parC* S80F point mutation (fluoroquinolone resistance).

The assembled genome of *S. aureus* strain MB02 has a total length of 2,750,504 bp with a GC content of 32.5% and an estimated genome coverage of 131×. The deposited assembly (GCA_049154425.1) consists of 76 contigs and 76 scaffolds with a contig N50 of 73.4 kb and L50 of 11. Genome annotation identified 2,876 total genes, including 2,741 protein-coding genes. Detailed genome statistics and features are presented in Table 1.

**TABLE 1: DATA:** availability, genome statistics, and features of *Staphylococcus aureus* MB02

Features	Value
Data availability	
GenBank assembly accession number	GCA_049154425.1
SRA accession number	SRR32454207
BioSample accession number	SAMN46967011
BioProject accession number	PRJNA1226935
Genome statistics	
Genome size (Mb)	2.8
Total ungapped length (Mb)	2.8
Number of scaffolds	76
Scaffold N50 (kb)	73.4
Scaffold L50	11
Number of contigs	76
Contig N50 (kb)	73.4
Contig L50	11
GC content (%)	32.5
Genome coverage (×)	131
Genome annotation	
Total genes	2,876
Protein-coding genes	2,741
Antimicrobial resistance genes	
Antimicrobial resistance determinants	*blaZ*, *blaI*, *blaR1*, *erm(C*), *aadD1*, *tet(38*), *tet(L*), *mupA*, *fusB*, *fosB*, *mepA*, *bleO*, *parC* S80F

## Data Availability

Raw sequencing reads are deposited in the NCBI Sequence Read Archive (SRA) under accession SRR32454207 (9). The genome assembly is available in GenBank under accession GCA_049154425.110, and the BioProject is PRJNA1226935 (11).
